# Post-ischemic Myocardial Inflammatory Response: A Complex and Dynamic Process Susceptible to Immunomodulatory Therapies

**DOI:** 10.3389/fcvm.2021.647785

**Published:** 2021-04-28

**Authors:** Niek J. Pluijmert, Douwe E. Atsma, Paul H. A. Quax

**Affiliations:** ^1^Department of Cardiology, Leiden University Medical Center, Leiden, Netherlands; ^2^Department of Surgery, Leiden University Medical Center, Leiden, Netherlands; ^3^Einthoven Laboratory for Experimental Vascular Medicine, Leiden University Medical Center, Leiden, Netherlands

**Keywords:** myocardial infarction, myocardial ischemia-reperfusion injury, inflammatory phase, reparative phase, innate immunity, chemokines, cytokines, inflammatory cells

## Abstract

Following acute occlusion of a coronary artery causing myocardial ischemia and implementing first-line treatment involving rapid reperfusion, a dynamic and balanced inflammatory response is initiated to repair and remove damaged cells. Paradoxically, restoration of myocardial blood flow exacerbates cell damage as a result of myocardial ischemia–reperfusion (MI-R) injury, which eventually provokes accelerated apoptosis. In the end, the infarct size still corresponds to the subsequent risk of developing heart failure. Therefore, true understanding of the mechanisms regarding MI-R injury, and its contribution to cell damage and cell death, are of the utmost importance in the search for successful therapeutic interventions to finally prevent the onset of heart failure. This review focuses on the role of innate immunity, chemokines, cytokines, and inflammatory cells in all three overlapping phases following experimental, mainly murine, MI-R injury known as the inflammatory, reparative, and maturation phase. It provides a complete state-of-the-art overview including most current research of all post-ischemic processes and phases and additionally summarizes the use of immunomodulatory therapies translated into clinical practice.

## Introduction

Ischemic myocardial injury causes decreased oxygen tension within the cell, subsequent degradation and loss of oxidative phosphorylation, and decreased generation of high-energy phosphates resulting in loss of membrane integrity (1). Irreversible cardiomyocyte injury, demonstrated by sarcolemmal disruption and presence of small amorphous densities in the mitochondria develops after 20–40 min of sustained severe ischemia (2). Cardiomyocyte cell death causes release of cardiomyocyte-specific proteins, such as myoglobin, cardiac troponin (cTn) T and I, creatine kinase MB (CK-MB), and creatine phosphokinase (CPK), which are used clinically as markers for early detection (3), especially by the use of high-sensitivity cTn assays nowadays (4). The dominant mechanism of cardiomyocyte death is coagulation necrosis, peaking after 12 h up to 4 days, with cell swelling and rupture of cell membranes resulting in extrusion of intracellular contents after passing the point of no return even though reperfusion is applied at a certain moment. This triggers an extensive inflammatory reaction activating reparative pathways ultimately contributing to mature scar formation (5). The other mechanism is apoptosis, peaking after 6–8 h, concerning programmed cell death, in particular, induced by reperfusion and also affecting non-infarcted areas of the LV wall and interventricular septum (6).

Myocardial infarct repair comprises three overlapping phases: the inflammatory, reparative, and maturation phase. The inflammatory phase contains activation of chemokine and cytokine cascades resulting in recruitment of local infiltration of leukocytes into the infarct area. Dead cells and matrix debris are cleared by neutrophils and macrophages. During the reparative or proliferative phase, activated mononuclear cell and macrophage subpopulations release cytokines and growth factors that recruit and activate predominantly myofibroblasts and vascular cells, while pro-inflammatory mediators are suppressed. Activated myofibroblasts abundantly produce extracellular matrix proteins, and an extensive microvascular network is formed with preservation of the structural integrity. In the maturation phase, reparative fibroblasts and vascular cells become apoptotic, and a cross-linked collagen-based scar is formed (7). This review addresses the post-ischemic inflammatory response in its entirety focusing on the role of innate immunity, chemokines, cytokines, and inflammatory cells in all three overlapping inflammatory, reparative, and maturation phases following experimental myocardial ischemia–reperfusion (MI-R) injury. The complexity of this process makes it amenable to pharmacologic interventions of which some has been successfully translated toward clinical practice as discussed in the final section.

## Myocardial Ischemia–Reperfusion Injury

As mentioned previously, early reperfusion by primary percutaneous coronary intervention (pPCI) is the recommended therapy in acute myocardial infarction (MI) since reperfusion restores oxygen and nutrient supply accentuating the post-ischemic inflammatory response and accelerating wound healing, making it a prerequisite for cardiomyocyte salvage (8). Even late reperfusion showed to be beneficial in humans, where it results in considerable myocardial salvage (9), as well as in animals, exhibiting permanent reduction of infarct expansion and ventricular remodeling (10). Paradoxically, restoration of myocardial blood flow comes at a price, as it initiates myocardial reperfusion injury by a series of events, which eventually provoke accelerated apoptosis (11).

There are four types of MI-R injury recognized. The first two are reversible and consist of reperfusion-induced arrhythmias, in particular, ventricular arrhythmias, which are usually self-terminating or effectively treated (12), and myocardial stunning, referring to reversible post-ischemic contractile dysfunction resulting from the detrimental effects of oxidative stress and intracellular calcium overload on the myocardial contractile apparatus (13). The other two are irreversible and are known as microvascular obstruction with reduced blood flow to the infarct zone despite patency of the infarcted-related artery (14, 15), and lethal reperfusion injury, which may account for up to 50% of the final infarct size (11).

The innate immune system is triggered following tissue injury resulting in the release of soluble inflammatory mediators soon after reperfusion, inducing an extensive inflammatory response as described above. Recruitment of inflammatory cells, oxidative stress, and endothelial barrier dysfunction compose myocardial healing in the early phase. Cytokines activate and recruit neutrophils to the injured infarct area, which cause direct injury to the endothelial cells via production of reactive oxygen species (ROS) (16), inflammatory cytokines, and adhesion molecules that facilitate binding of leucocytes and platelets. Other mediators of myocardial reperfusion injury are the opening of the mitochondrial permeability transition pore, cardiomyocyte calcium overload with hypercontracture, and intracellular pH changes resulting in a wave front of reperfusion injury with a strict therapeutic window (17). Reperfusion also induces and aggravates apoptosis (6). Through binding and ingestion of dying cells, myeloid cells can markedly influence immune responses. MI-R-induced apoptosis results in changes in cellular structures including loss of the asymmetric distribution of plasma membrane phospholipids (18).

### Innate Immunity

**Figure 1:**
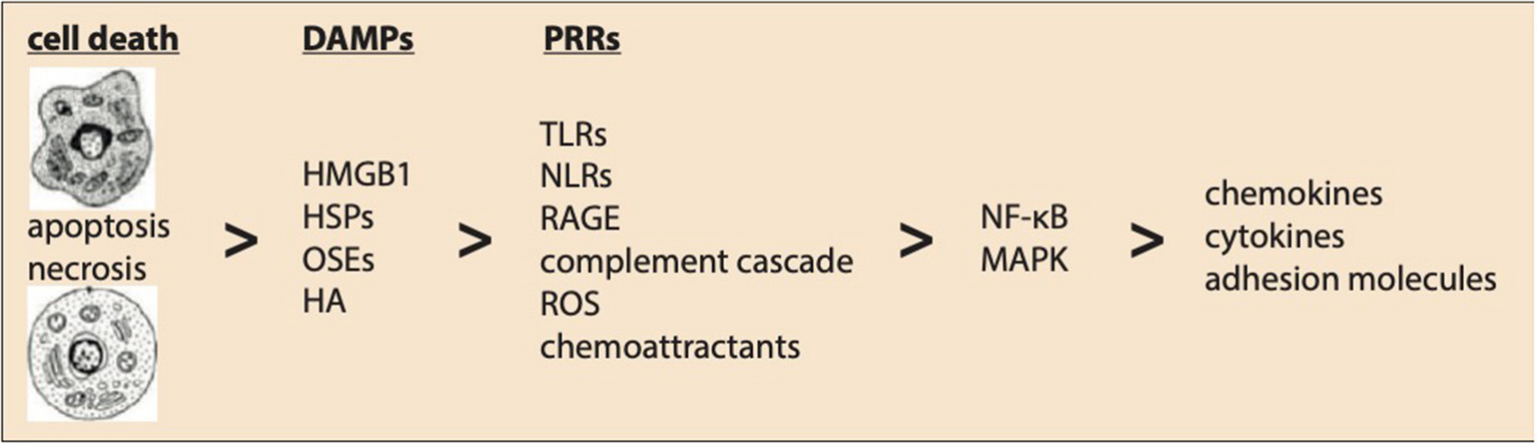
Innate immunity following myocardial ischemia–reperfusion injury. Ischemic-induced myocardial cell death activates innate immunity. Expression of endogenous ligands upon reperfusion are known as danger-associated molecular patterns (DAMPs) including HMGB1, high-mobility group box-1; HSPs, heat shock proteins; OSEs, oxidation-specific epitopes; HA, hyaluronic acid. These DAMPs are recognized by pattern recognition receptors (PRRs) like TLRs, NLRs, RAGE, complement cascade, reactive oxygen species (ROS), and chemoattractants. Finally, activation of the nuclear factor (NF)-κB and mitogen-activated protein kinase (MAPK) pathways induce the expression of pro-inflammatory chemokines, cytokines, and adhesion molecules regulating a complex post-ischemic inflammatory response.

Cell death induced by necrosis causes a release of intracellular contents and triggers an inflammatory response by activating the innate immune system (Figure 1). Expression of endogenous ligands upon reperfusion are judged as “danger signals.” These danger-associated molecular patterns (DAMPs) are recognized by both signaling and endocytic pattern recognition receptors (PRRs), such as Toll-like receptors (TLRs), expressed by cells of the innate immune system. Activation of inflammatory pathways by activation of, e.g., TLR-mediated pathways, chemoattractants, the complement cascade, and ROS as a result of MI induces the expression of a large panel of pro-inflammatory genes driven by the activation of mitogen-activated protein kinases (MAPKs) and especially nuclear factor (NF)-κB (7).

#### Danger-Associated Molecular Patterns

The innate immune system can be triggered by endogenous, non-pathogenous signals, referred to as DAMPs (19). These endogenous ligands released in response to MI include high-mobility group box-1 (HMGB1), heat shock proteins (HSPs) and S100-proteins, nuclear and mitochondrial DNAs, RNAs, adenosine triphosphate (ATP), low molecular weight hyaluronic acid, and fibronectin fragments (20). Oxidation-specific epitopes (OSEs) can act as endogenous DAMPs as well (21).

HMGB1, the best characterized danger signal, is a key initiator of inflammatory injury following myocardial ischemia controlled by action mechanisms involving TLRs (22, 23) and RAGEs, the receptor for advanced glycation end products (22). HMGB1 mediates MI-R injury by activation of inflammatory pathways (24), but also confers favorable effects by enhancing angiogenesis, reducing infarct size and improving cardiac function following MI (25). Also, HSPs showed both beneficial and detrimental effects following myocardial ischemia. For instance, HSP20 and HSP70 protect cardiomyocytes against MI-R injury in rodents, with improved recovery of cardiac contractile performance and decreased infarct size (26). However, HSP60 has strongly been associated with the development of ischemic heart failure (27), and HSP60-mediated activation of TLR4 induced myocardial apoptosis and cytokine expression following MI-R injury (28). Also, low molecular weight hyaluronic acid and fibronectin are capable of activating the innate immune system via TLRs (29, 30).

#### Toll-Like Receptors

TLRs are highly conserved transmembrane receptors and comprise the major group of PRRs, and recognize a variety of DAMPs and pathogen-associated molecular patterns (PAMPs). The TLR family consists of 13 different receptors, classified into the plasma membrane or endosomal localized TLR subfamilies, triggering the innate immune system upon binding of their ligands leading to the dimerization of the cytosolic Toll/interleukin-1 receptor (TIR) domain, ultimately activating nuclear factor (NF)-κB and MAPK pathways to upregulate pro-inflammatory mediators (31, 32). Myocardial infarction induced local TLR4 expression (33), and upregulated TLR4 in cardiomyocytes that exacerbated heart failure (34). Furthermore, TLR4 deficiency resulted in decreased MI-R injury (35), restricted cardiomyocyte apoptosis (23), and attenuated adverse cardiac remodeling resulting in improved LV function (36). In addition, administration of a chemical TLR4 inhibitor reduced the recruitment of Ly-6C^hi^ monocytes accompanied by impaired NF-κB activation and cytokine expression. This resulted in decreased infarct size (37). TLR2 deficiency resulted in reduced LV dilation and improved LV function due to decreased fibrosis in the non-infarcted area (38), and antagonizing TLR2 reduced infarct size after MI-R injury (39). Although less extensive, other TLRs have been analyzed as well following MI-R injury. Signaling of TLR3-TIR domain-containing adaptor inducing IFN-β-mediated transcription factor (Trif) represents an injurious pathway, since TLR3-Trif deletion reduced infarct size and improved LV function probably by mediating myocardial apoptosis (40). In addition, administration of the TLR9 ligand activated the PI3K/Akt signaling pathway conferring cardioprotective effects by induction of TLR9 tyrosine phosphorylation and association with the p85 subunit of PI3K (41). Taken together, TLRs are promising therapeutic targets, and several TLR (ant)agonists already have been developed and investigated following MI-R injury (42, 43).

#### Nucleotide-Binding Oligomerization Domain-Like Receptors

NLRs are a group of intracellular PRRs with over 20 members. NLRs consist of a pyrin (PYD) or caspase recruitment (CARD) domain at the N-terminus combined with a central nucleotide binding (NACHT) followed by a C-terminal leucine-rich repeat (LRR) domain. The inflammasome comprises an activated NLR protein, the adaptor apoptosis speck-like protein containing a caspase-recruitment (ASC) domain, and procaspase-1 (44). After an MI, the major components of the NLRP3 inflammasome are upregulated and/or activated in leukocytes, fibroblasts, and endothelial cells, as well as in border zone cardiomyocytes, resulting in increased interleukin (IL)-1β and IL-18, its cytokine end products. Targeted gene disruption (45) and antibody neutralization (46) have been shown to reduce infarct size after MI-R injury, whereas NLRP3 deficiency (47) and selective NLRP3 inhibition (48) exhibited additional preservation of cardiac function.

#### Receptor for Advanced Glycation End Products

The receptor for advanced glycation end products also serves as a multiligand PRR, which triggers a number of cytosolic signaling pathways, including NF-κB and MAPK-dependent inflammatory genes (49). RAGE deficiency protected against MI-R injury resulting in improved cardiac function and remodeling, while RAGE-mediated signaling is essential for circulating cells to migrate to the myocardium and exert detrimental effects (50). Accordingly, co-treatment of soluble RAGE and siRNA RAGE exhibited synergistic cardioprotective effects following MI-R injury (51), and modulation of the AGE-RAGE/MAPK pathways resulted in improved cardiac function by attenuating MI-R injury (52).

#### Complement Cascade

Involvement of the complement system in myocardial ischemia was first demonstrated almost five decades ago (53), and activation has been suggested to extend ischemic injury (54). The complement cascade operates on the transition from innate to adaptive immunity and is activated through three different mechanisms defined as the classical, alternative, and lectin pathways. All three lead to cleavage of C3 with subsequent activation of C5 and formation of the membrane attack complex (MAC) (55). It consists of numerous plasma and cell membrane proteins, which are activated following binding of antibodies to PAMPs and release of DAMPs in response to tissue injury. Also, C-reactive protein and IgM play an important role since they are co-localized with activated complement in human infarcted myocardium (56). Activation causes opsonization of pathogens, phagocytosis, augmentation of inflammation, and direct cell lysis, which is regulated by many regulatory proteins (57).

All three pathways are involved in the pathogenesis of MI-R injury, by activation-mediated neutrophil and monocyte recruitment in the ischemic myocardium especially in the first hours of reperfusion (58). The lectin pathway, initiated by recognition of the circulating pathogen recognition molecule mannose-binding lectin (MBL), seemed to be the major contributor. Complement inhibition by antibodies against MBL reduced neutrophil infiltration and attenuated pro-inflammatory gene expression (59). In addition, MBL deficiency with concomitant active alternative and classical pathways protected against MI-R injury with resultant preservation of cardiac function (60).

Blocking the complement cascade may reduce myocardial injury, which has been accomplished by consumptive depletion with cobra venom factor (61), antibody-induced inhibition (62), genetic deficiency (63), or ablated plasma activation (64) of individual complement components such as C5, or by infusion of modified native components that block activation like soluble complement receptor type 1 (sCR1) (65) and C1-esterase inhibitor (C1-INH) (66). Despite these promising results in animal models, clinical studies concerning C5 inhibition in humans have been performed with disappointing results (67).

#### Activation of NF-κB

Induction of pro-inflammatory mediators in ischemic myocardium is controlled by activation of the NF-κB system, a central transcriptional effector of inflammatory signaling, triggering transcription of various genes including inflammatory cytokines, CXC and CC chemokines, and adhesion molecules upon MI (68). The family of transcription factors consists of at least five subunits, RelA (p65), RelB, c-Rel, p50, and p52 (69). NF-κB signaling in the heart has been controversial and seems to regulate three genetic programs depending on the timing and cellular activation, known as acute cytoprotection following hypoxia/ischemia, chronic cytotoxicity generated by a prolonged inflammatory response, and hypertrophy (68).

Pharmacological blockade of inhibitor of NF-κB (IκBα) (70) and inhibitor of NF-κB kinase subunit β (IKKβ) (71), cardiomyocyte-specific NF-κB deletion (72), and blocking of the inflammatory gene activation (73) decreased infarct size, reduced inflammatory responses, and improved cardiac function following MI-R. However, several studies reported conflicting results endorsing the various cellular processes and molecular pathways affected by the NF-κB system, which are also strictly dependent on the cellular context and timing of activation since prolonged NF-κB activation promoted heart failure by provoking chronic inflammation (68).

#### Chemokines

**Figure 2:**
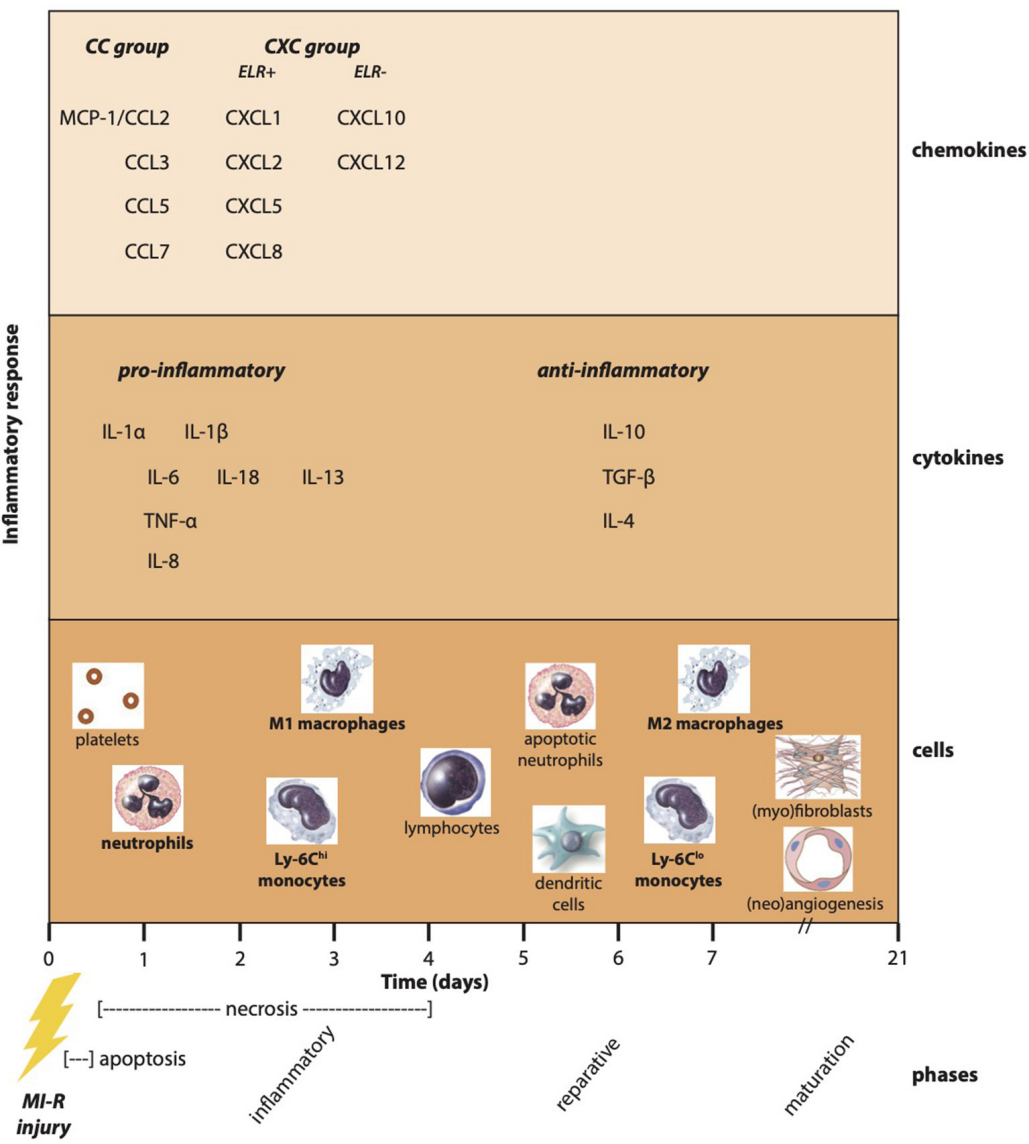
Post-ischemic inflammatory response. Myocardial infarction especially followed by reperfusion results in a complex post-ischemic inflammatory response encompassing a multifactorial and dynamic process. Apoptosis and necrosis activate the innate immune system resulting in the upregulation of pro-inflammatory chemokines and cytokines. As a result, inflammatory cells are attracted to the ischemic myocardium known as the inflammatory phase. In addition, the production of anti-inflammatory cytokines causes a transition toward the reparative phase accompanied by reparative monocyte and macrophage subtypes. Finally, (myo)fibroblasts and angiogenic cells ensure the formation and maturation of myocardial scar tissue.

Following MI, a variety of pro-inflammatory chemokines and cytokines are upregulated as a result of activation of the innate immune system (Figure 2). Chemokines are divided into subfamilies on the basis of the number and sequential relationship of their conserved cysteine residues (CC, CXC, CX3C, and XC) and play a critical role in basal and inflammatory leukocyte trafficking (74). Functionally, they can be divided in homeostatic chemokines, which are constitutively expressed in certain tissues and execute basal leukocyte trafficking, and inducible or inflammatory chemokines, which are markedly upregulated following tissue injury and actively induce leukocyte recruitment (75). Expression of chemokines is managed by activation of TLR-mediated pathways, the complement cascade, ROS generation, and the NF-κB system following myocardial ischemia, while reperfusion accentuates chemokine expression (76).

#### CC Chemokines

CC chemokines are the largest and most distinct subfamily and function as potent mononuclear cell chemoattractants. The monocyte chemoattractant protein (MCP)-1/chemokine ligand (CCL)2 is rapidly upregulated in the ischemic myocardium and operates as a potent chemoattractant to mononuclear cells following reperfusion (77). In addition, MCP-1 deficiency attenuated post-ischemic LV remodeling as a result of a prolonged inflammatory phase with decreased and delayed macrophage infiltration (78). However, in the absence of reperfusion, cardiac overexpression of MCP-1 induced macrophage infiltration, neovascularization, myocardial IL-6 secretion, and myofibroblast accumulation, which prevented LV dysfunction and adverse remodeling after MI (79). Furthermore, administration of a CCL2 competitor reduced monocyte recruitment, which attenuated MI-R injury (80), and pharmacological blockage of the CCL5-CXCR4 interaction impaired the inflammatory response resulting in a reduced infarct size and preserved cardiac function (81). Similar results were observed with anti-CCL5 antibody therapy following permanent ischemia as a result of impaired infiltration of mononuclear cells (82). Absence of the chemokine receptor CCR1 resulted in reduced functional impairment and structural remodeling due to an abolished early inflammatory recruitment of neutrophils accompanying improved tissue healing (83). More recently, CCR9 deficiency was demonstrated to reduce mortality as a result of a decreased infarct size and limited adverse LV remodeling associated with an attenuated inflammatory response (84).

### CXC Chemokines

CXC chemokines that contain an ELR motif are critically involved in chemotactic recruitment of neutrophils, and those lacking the ELR motif regulate the recruitment and activation of lymphocytes. From the ELR-containing CXC chemokines, CXCL8/IL-8 is upregulated in experimental MI-R and participates in neutrophil-mediated myocardial injury (85, 86). IL-8 induces chemotaxis and granulocyte recruitment, enhances β2 integrin-dependent cellular adhesion (87), and inhibition with a neutralizing monoclonal antibody reduced the degree of necrosis after MI-R independent of neutrophil infiltration (88). Other ELR-containing CXC chemokines, such as growth-regulated oncogene (GRO)-α/KC/CXCL1, macrophage inflammatory protein (MIP)-2/CXCL2, and lipopolysaccharide-induced chemokine (LIX)/CXCL5, induced neutrophil chemotaxis and activation, and are expressed by infiltrated inflammatory cells and ischemic myocardium following MI-R injury (89). In addition, deficiency of the chemokine receptor CXCR2 showed predominant cardioprotective effects with reduced infarct size and recruitment of inflammatory cells after MI-R injury (90). Also, pharmacological inhibition by a chemokine-binding protein of CXC chemokines, such as CXCL1 and CXCL2, reduced infarct size by preventing neutrophil recruitment and ROS production (91).

Of the ELR-negative CXC chemokines, interferon-inducible protein (IP)-10/CXCL10 peaks in the early reperfusion phase and regulates the cellular composition attenuating adverse remodeling, since IP-10 deficiency resulted in a hypercellular early reparative response with intense infiltration of myofibroblasts suggesting the exertion of anti-fibrotic actions following myocardial ischemia (92). Finally, stromal cell-derived factor (SDF)-1/CXCL12 may recruit progenitor cells with angiogenic potential, contributing to neovascularization of the scar (93), and is upregulated after MI regulating therapeutic stem cell homing to ischemic myocardium, which resulted in preserved cardiac function (94). Improved cardiac function after myocardial ischemia was attributed to increased angiogenesis and decreased scar formation as a result of SDF-1α treatment (95), and the protective effect was blocked by the administration of a selective antagonist following MI-R injury (96). However, conflicting observations have been reported following MI-R with detrimental effects of CXCR4-CXCL12 overexpression possibly as a result of enhanced inflammatory cell recruitment, and activation of cell death or apoptosis (97). These conflicting results again reflect the complexity of the reparative process with multifunctional, pleiotropic, and context- and timing-dependent actions of chemokine signaling.

### Cytokines

Following acute MI, various inflammatory cytokines are secreted by circulating inflammatory cells and cardiac resident cells, amplifying the pro-inflammatory response to acute ischemia–reperfusion injury by moderating the local recruitment of inflammatory cells (Figure 2). Pro-inflammatory cytokines tumor necrosis factor (TNF)-α, IL-1, and IL-6 have consistently been found to be induced and released following MI (98, 99), whereas IL-10 and transforming growth factor (TGF)-β exerted anti-inflammatory effects (5, 98).

#### Pro-inflammatory Cytokines

Induction and release of pro-inflammatory cytokines play an important role in mediating chemokine upregulation in the injured myocardium. TNF-α stimulates expression of other pro-inflammatory cytokines, chemokines, and adhesion molecules by leukocytes and endothelial cells, and regulates extracellular matrix metabolism by decreasing collagen synthesis and activating matrix metalloproteinase (MMP) activity (100). Targeted TNF-α overexpression caused adverse cardiac remodeling provoked by progressive cardiomyocyte apoptosis (101). Following MI-R injury, TNF-α deficiency exhibited attenuated chemokine expression and NF-κB activation in the infarcted heart resulting in reduced infarct size and improved cardiac function (102). Additional remote ischemic preconditioning prior to reperfusion attenuated the inflammatory response causing decreased levels of TNF-α and IL-1β accompanied with an improved LV function (103). Pharmacological treatment with morphine following MI-R injury was shown to decrease circulating TNF-α levels and reduce infarct size with concomitant improvement of LV function (104). Furthermore, G protein-coupled receptor kinase 2 (GRK2) deficiency resulted in decreased TNF-α expression and fibrosis resulting in reduced infarct size and preserved cardiac function (105). In experimental MI, TNF-α deficiency protected against myocardial rupture and chronic LV dysfunction by inhibiting abundant inflammation, matrix and collagen degradation, and apoptosis (106). In contrast, protective effects of TNF-α signaling are described as well, since neutralizing TNF-α with adenoviral TNFR1 exhibited detrimental effects by promoting ventricular rupture and exacerbating ventricular dysfunction and remodeling following MI (107). Complexity of cytokine expression regarding therapeutic interventions remains a challenge and influences its effects, which might partially depend on blockage of specific receptors since TNF-α effects was shown to be toxic via TNFR1 and protective via TNFR2 (108, 109).

Neutralization of IL-1 by genetic overexpression of IL-1 receptor antagonist protected the myocardium from MI-R injury by attenuating the inflammatory response associated with decreased apoptosis (110), and reduced inflammatory markers in patients with acute coronary syndrome (111), but exerted no functional improvement (112). Renal sympathetic denervation resulted in a reduced circulating production of pro-inflammatory cytokines IL-1 as well as IL-6 and TNF-α, which was associated with decreased circulating inflammatory cells, reduced infarct size, and improved LV ejection fraction following MI-R injury (113). Administration of a caspase-1 inhibitor combined with a P2Y_12_ antagonist decreased circulating IL-1β, reducing infarct size and preserving LV-function (114). In addition, treatment with an IL-1 receptor (IL-1R) antagonist as well as IL-1RI deficiency exhibited decreased neutrophil and macrophage infiltration of the injured myocardium with reduced chemokine and cytokine expression subsequently resulting in reduced cardiomyocyte apoptosis and an attenuated fibrotic response preserving adverse remodeling (115, 116).

Synthesis of IL-6 is induced rapidly in the ischemic-reperfused myocardium (117), and IL-6 has been associated with acute coronary syndrome (118). In mendelian randomization studies, IL-6R inhibition resulted in reduced cardiovascular events suggesting a novel therapeutic approach to prevent coronary heart disease (119). In addition, administration of tocilizumab, an IL-6R antagonist, in patients suffering a non-ST-elevation MI attenuated the inflammatory response with reduced levels of high-sensitivity C-reactive protein and cTnT (120). However, pre-clinical studies reported conflicting results, as IL-6 deficiency did not affect infarct size, LV function or remodeling, and survival after unreperfused MI, which was explained by a compensatory role of other mediators (121) but, on the other hand, reduced infarct size following MI-R injury (122). Furthermore, administration of the IL-6R antibody MR16-1 after MI suppressed myocardial inflammation causing impaired LV-remodeling and improved LV contractile function (123), but worsened LV remodeling following MI-R injury (124). Regarding pharmacological treatment, administration of bisoprolol conferred cardioprotective effects following MI-R injury by suppressing both IL-6 as TNF-α secretion and attenuating the unfolded protein response, thereby reducing infarct size and improving post-ischemic cardiac function (125). Linagliptin, a dipeptidylpeptidase (DPP)-4 inhibitor, attenuated the increase in IL-6 as well as IL-1β and TNF-α following MI-R injury, which was associated with a limited infarct size and improved ejection fraction (126).

Overall, attenuating the effects of pro-inflammatory cytokines seemed cardioprotective; however, conflicting results suggest them to exert diverse biological effects and emphasize the complex and pleiotropic cytokine actions in biological processes.

#### Anti-inflammatory Cytokines

Inhibitory mediators, such as IL-10, TGF-β, or lipid-derived substances may be the predominant mechanism for deactivation of chemokine signals. They exert pleiotropic effects in regulating the immune response and are leading in mediating the post-ischemic reparative response (127).

IL-10 is predominantly expressed by activated T-lymphocytes and stimulated monocytes, and possesses potent anti-inflammatory properties. Induction of IL-10 coincides with the suppression of IL-6 formation in macrophages and monocytes and further inhibits production of IL-1α, IL-1β, TNF-α, and IL-8, suppressing the inflammatory response. Furthermore, it stabilizes the matrix by decreasing metalloproteinase biosynthesis (128). IL-10 is upregulated in reperfused myocardium, and neutralizing IL-10 activity caused reduced expression of tissue inhibitor of metalloproteinases (TIMP)-1, suggesting IL-10 to contribute to stabilization of the matrix (129). Following MI-R injury, IL-10 deficiency resulted in increased fibrosis and apoptosis (130) and an enhanced inflammatory response, associated with increased infarct size and mortality (131). In addition, administration of recombinant IL-10 following MI suppressed the inflammatory response and contributed to improved LV function and remodeling by inhibiting fibrosis and enhancing capillary density (132). Pharmacological treatment with colchicine before (133) or a selective β_2_ adrenergic receptor agonist during (134) MI-R injury increased systemic IL-10 levels exhibiting a cardioprotective effect with a reduced infarct size. However, conflicting evidence regarding the role of IL-10 in the resolution of the inflammatory response following MI-R exists, as IL-10 deficiency also showed timely repression of pro-inflammatory cytokines and chemokine mRNA synthesis with a similar course of neutrophil infiltrate resolution, indicating the involvement of multiple overlapping modulating mechanisms (135).

Cell proliferation, differentiation, and apoptosis as well as modulation of the immune response are all regulated by TGF-β. Because of its anti-inflammatory and fibrogenic properties, it affects cardiac remodeling by mediating transition from inflammation to fibrosis (136). Upon TGF-β stimulation, myofibroblast transdifferentiation is induced, and the activity of proteases that degrade the extracellular matrix (ECM) is suppressed (137). Following myocardial ischemia TGF-β is increased, of which TGF-β1 and TGF-β2 isoforms are upregulated within the first few days in contrast to the delayed and prolonged expression of the TGF-β3 isoform showing a positive correlation with parameters of the ECM metabolism (138). TGF-β is predominantly located in the infarct border zone activated by the induction of thrombospondin (TSP)-1 following MI-R injury (139). The signaling pathway is regulated through Smad proteins (140) of which Smad3 signaling seemed to be critical in cardiac remodeling (141).

Post-ischemic treatment with TGF-β prevented severe cardiac injury, presumably by attenuating the deleterious effects of pro-inflammatory cytokines (142). Loss of the anti-inflammatory mechanism of growth differentiation factor (GDF)-15, a TGF-β-related cytokine and inhibitor of leukocyte integrin activation, resulted in fatal cardiac rupture after MI (143). Moreover, prevention of myeloid-epithelial-reproductive tyrosine kinase (MerTK) cleavage following MI-R injury resulted in secretion of TGF-β and IL-10, which was associated with a reduced infarct size and improved systolic function (144). However, reducing TGF-β activity by inhibiting the TGF-β type I receptor, attenuated systolic dysfunction and LV remodeling (145), and gene therapy against TGF-β mitigated LV remodeling as well (146). Also, inhibition of TGF-β expression due to a combination therapy of angiotensin-converting enzyme (ACE) inhibitor and angiotensin receptor blocker attenuated cardiac fibrosis and remodeling (147). Furthermore, targeted disruption of TGF-β signaling by conditional deficiency of the TGF-β receptor 1 or 2 resulted in a marked decline in neutrophil recruitment and upregulation of protective cardiokines protecting from LV wall rupture (148). Finally, Smad3 deficiency following MI-R injury resulted in reduced interstitial fibrosis and attenuated cardiac remodeling due to abrogation of the fibrogenic TGF-β responses (149). Discrepancies might be explained by a specific time window of action since activation of TGF-β signaling seemed protective in the early post-ischemic phase, but caused adverse LV remodeling and dysfunction when expression persists over time (150).

The inhibitory cytokines exert pleiotropic effects regarding their inflammatory and reparative properties, which can be either stimulatory or inhibitory, depending on the state of cellular differentiation, microenvironmental cues, and the cellular tissue origin. The related complexity has hampered the translation from attractive hypothesis toward novel therapeutic strategies exhibiting functional improvement in experimental MI models.

### Inflammatory Cells

Acute MI results in a complex inflammatory response associated with induction of endothelial adhesion molecules and enhanced permeability of the microvasculature. Upregulated chemokines and cytokines cause extravasation of activated blood-derived cells into the infarcted area (Figure 2), which play an important role in regulating the reparative response and are possible targets for cardioprotective therapies (151). Single-cell RNA-sequencing following unreperfused MI of the non-cardiomyocyte fraction revealed over 30 cell subtype populations, which highlighted post-ischemic non-linear dynamics in myeloid and fibroblast lineages crucial for understanding of cardiac homeostasis, inflammation, fibrosis, repair, and regeneration (152).

At first, platelets are recruited into the injured myocardium representing an important linkage between tissue injury and repair (153). They target monocytes, macrophages, and T-cells by releasing mitogenic factors, recruit leukocytes by production of cytokines and chemokines, and trigger complement activation (154, 155). Following MI-R injury, platelet-derived serotonin induced neutrophil degranulation with release of myeloperoxidase, which was abolished after additional genetic or pharmacological depletion preserving cardiac function (156). In addition, targeting activated platelets with a single-chain antibody-CD39 (Targ-CD39) resulted in attenuated post-ischemic inflammation, reduced infarct size, and increased neovascularization resulting in a remarkable restoration of ejection fraction and strain rate (157).

Briefly, MI triggers a biphasic reaction of inflammation and remodeling, with immediate influx of neutrophils and CCR2^+^Ly-6C^hi^ pro-inflammatory M1 monocytes/macrophages in the immediate phase, which phagocytose debris and secrete pro-inflammatory cytokines IL-1β, IL-6, and TNFα. After around 3 days transition to the reparative phase is achieved in which accumulated Ly-6C-F4/80^hi^ M2 macrophages secrete anti-inflammatory cytokines IL-10 and TGF-β (158).

### Inflammatory and Reparative Phases

#### Neutrophils

Prior to MI, neutrophils as well as monocytes already circulate in the blood in a steady state. In response to experimental MI, neutrophils, which are efficient phagocytes that engulf and degrade microorganisms using oxidative and non-oxidative mechanisms, are migrated into the injured myocardium as the first line of defense to phagocytose death myocardial cells (159). In the clinical setting, elevated neutrophil as well as leukocyte counts following primary PCI in ST-segment elevation MI are directly related to infarct size and LV ejection fraction and are independent predictors of cardiovascular outcomes (160). Neutrophils infiltrate the injured myocardium within hours, peaking at 1–3 days and primarily tend to target the border zone showing accentuated accumulation at reperfusion (161). They are recruited to the injured myocardium by a high concentration of chemotactic factors, such as MIP-2α, IL-8(/CXCL8), CXCL1(/GRO-α/KC), complement 5a, and leukotriene B4. Migration from the circulation into the injured myocardium depends on binding to P-selectin, E-selectin, intercellular adhesion molecule (ICAM)-1, and vascular cell adhesion molecules (162). Until recently, it was believed that cardiac neutrophils demonstrate a pro-inflammatory N1 phenotype, being predominant in the infarct area correlating with wall thinning, and an anti-inflammatory N2 phenotype, increasing over the course of MI (163). Excessive infiltration and/or delayed removal may exacerbate injury by intensifying the inflammatory response. Novel insights show a more complex concept of temporal cardiac neutrophil heterogeneity in experimental MI. Single-cell RNA sequencing combined with surface epitope detection demonstrated more complex neutrophil dynamics with two major subsets. The SiglecF^lo^ neutrophils, resembling circulating blood neutrophils, and the SiglecF^hi^ neutrophil subset, exclusively found in the heart, are characterized by specific effector functions as phagocytosis and production of ROS (164).

Targeting neutrophils to reduce accumulation into the injured myocardium has been shown to reduce infarct size and mostly improved cardiac function following neutralization of the receptor activator of NF-κB ligand (165), plasminogen activator inhibitor-1 deficiency (166), brahma-related gene 1 (BRG1) deficiency (167), pharmacological inhibition, as well as deficiency of transient receptor potential melastatin 2 (TRPM2) (168), or proteasome-mediated IκBα inhibition (169). Furthermore, inhibition of neutrophil activity exposed beneficial effects with a reduced infarct size as a result of pretreatment with a dual cyclooxygenase–lipoxygenase blocking agent before MI-R injury (170). Improved LV function and attenuated adverse remodeling were shown following administration of an inhibitor of the triggering receptor expressed on myeloid cells-1 (TREM-1) (171), a S100A8/A9 blocker (172), or myeloperoxidase inhibitor (173) early after initiating reperfusion. In addition, neutralization of L-selectin and P-selectin with monoclonal antibodies attenuated neutrophil accumulation and reduced myocardial necrosis after MI-R injury (174, 175). However, combined P-selectin and ICAM-1 deficiency demonstrated impaired neutrophil trafficking but did not affect infarct size in response to MI-R (176). Moreover, inhibition or loss of the CD11/CD18-integrin receptor, which allows neutrophil binding and transmigration, resulted in diminished neutrophil accumulation accompanied by a reduced infarct size and preserved cardiac function after experimental MI-R injury (177–179). Disappointingly, clinical studies targeting CD11/CD18 subunits did not report any cardioprotective effect following acute MI, endorsing its challenging aspects (180, 181). Finally, complete neutrophil depletion was shown to be detrimental, since neutrophils are crucially involved by polarizing macrophages toward a reparative phenotype, emphasizing the importance of carefully balanced novel anti-inflammatory therapeutic strategies (182).

#### Mononuclear Cells

Following experimental MI-R injury, mononuclear cells (monocytes and lymphocytes) are rapidly recruited into the ischemic myocardium, visualized by real-time *in vivo* imaging (183), due to various monocyte chemoattractants driven by MCP-1 (78). Monocytes and macrophages have a critical role in post-ischemic cardiac repair, since they promote both injury and repair, and express wide heterogeneity and dynamics during the immediate inflammatory response. As a result of cardiomyocyte injury, monocytes and monocyte-derived macrophages infiltrate the heart and largely replace tissue-resident macrophages. Cardiac tissue-resident macrophages originate from CX3CR1+ progenitors (184), are self-renewing and long-lived (185), and display an anti-inflammatory F4/80^hi^Ly-6C^lo^ or M2 phenotype (186). A combination of single-cell RNA sequencing with genetic fate mapping of the healthy adult myocardium revealed four transcriptionally distinct cardiac macrophage subsets, which are, on the one hand, maintained independent of blood monocytes, or on the other hand, partially or fully replaced by monocytes. Following myocardial injury, resident cardiac macrophages, suppressed by peripherally CCR2+ monocyte-derived macrophages, accounted for only 2–5% of the cardiac macrophages within the infarct area (187). Resident cardiac macrophages undergo continuous and distinct transcriptomic changes (186), and their depletion promoted adverse remodeling and impaired cardiac function (187).

Of the monocytes, the pro-inflammatory (Ly-6C^hi^) monocytes migrate to the site of injured myocardium at first, peaking 3 days after MI, which express TNF-α and IL-1β, produce proteolytic enzymes, and secrete MMPs. They exhibit rapid kinetics with a relatively short circulating life-span (188), and differentiate, in the presence of IFN-γ or lipopolysaccharide (LPS), into activated pro-inflammatory M1 (CCR2+) macrophages secreting large amounts of pro-inflammatory mediators (189). In addition, nuclear receptor subfamily 4, group a, member 1 (Nr4a1) seems critical for Ly-6C^hi^ monocytes to exert either inflammatory or reparative effects following unreperfused MI. Although differentiated cardiac macrophages depend on Nr4a1 to limit inflammation, the apoptotic or proliferation processes seem unaffected (190). Second, after about 7 days, the reparative (Ly-6C^lo^) monocytes, also suggested to directly correspond with F4/80^hi^Ly-6C^lo^ macrophages originated from Ly-6C^hi^ monocytes (190, 191), become predominant, which differentiate, in the presence of IL-4 and IL-10, toward a reparative M2 (CCR2–) phenotype, promoting the healing response, and contributing to angiogenesis and scar maturation (189). Tissue-resident CCR2+ and CCR2– macrophages differentially regulated cardiac mobilization and recruitment of peripheral monocytes where depletion of tissue-resident CCR2+ macrophages substantially reduced the recruitment of recipient monocytes and neutrophils accompanied by an improved LV systolic function following murine MI-R injury (185). In the clinical setting, higher levels of pro-inflammatory monocytes were associated with severe myocardial injury and poor functional outcome following acute MI (192, 193). Furthermore, the macrophage migration inhibitory factor (MIF) seemed to exert pleiotropic effects following MI-R injury. By activating the cardioprotective AMP-activated protein kinase (AMPK) pathway, it seemed beneficial, on the one hand (194), but MIF deficiency, on the other hand, turned out to protect the heart by suppressing the inflammatory response as well (195). The compartmentalized and opposing effects are largely mediated by CXCR2, where CXCR2-bearing inflammatory cells revealed to be detrimental by increasing monocyte infiltration (196). Efferocytosis of apoptotic cardiomyocytes by macrophages suppressed expression of pro-inflammatory mediators, which may drive resolution of inflammation (197).

Attenuating the pro-inflammatory phase by targeting Ly-6C^hi^ monocytes or M1 macrophages has been shown cardioprotective following reduction of interferon regulatory factor 5 (IRF5) expression (198), injection of phosphatidylserine (PS)-presenting liposomes (199), or irbesartan-nanoparticles (200), as well as administration of annexin A5 (AnxA5) (201) or phosphorylcholine monoclonal immunoglobulin G antibodies (202), and CCR-2 silencing (203). In addition, expediting the differentiation from reparative Ly-6C^lo^ monocytes toward M2 macrophages by administration of pioglitazone-nanoparticles (204) or topiramate (205), T_reg_-cell activation (206), and silencing the collapsin response mediator protein-2 (CRMP2) (207) limited adverse remodeling. Moreover, exacerbation of the post-ischemic inflammatory response by administering 2-arachidonolyglycerol resulted in increased neutrophil and monocyte counts associated with an increased infarct size and worsened cardiac function (208). Inhibition of the reparative phase finally, by selective depletion of M2 macrophages as a result of deficiency of the kinase tribbles homolog 1 (TRIB1) caused an increased risk of cardiac rupture following MI, emphasizing their role in regulation of fibroblast activation (209).

Other leukocytes, like lymphocytes, dendritic cells, and mast cells invading the injured myocardium includes smaller numbers, but may have an important role in regulating infarct healing and the myeloid response. CD4+ T-lymphocytes was shown to become activated after MI and improved beneficial remodeling and survival (210). Lymphopenia, primarily due to loss of T cells in patients with an acute MI, was correlated with substantial microvascular obstruction (211). Furthermore, T_reg_-cells are known to attenuate the innate immune response and inducing T_reg_-cells by injection of tolerogenic dendritic cells (tDCs) elicited an inflammatory-to-reparative macrophage shift yielding improved remodeling associated with preservation of LV function and increased survival (212). However, CD4+ T-cell deficiency also showed to reduce infarct size and preserve ejection fraction following MI-R injury (213), suggesting different roles of T-cells regarding infarct remodeling compared with MI-R injury. B-lymphocytes peak 5 days after acute MI and trigger monocyte mobilization, which deteriorated LV function (214). Following MI, granulocyte—macrophage colony-stimulating factor (GM-CSF)-producing B cells, dendritic cells, and T cells expand in pericardial adipose tissue, at which both B-cell depletion and GM-CSF blockade, as well as removal of pericardial adipose tissue reduced fibrosis and preserved cardiac function (215).

Dendritic cells are antigen-presenting cells contributing to innate immunity and providing a link to the adaptive immune system. Following MI-R injury, they migrate and accumulate in the injured myocardium, and deficiency resulted in increased pro-inflammatory cell and cytokine recruitment with worsened adverse remodeling suggesting immunoprotective effects (216). In addition, exosomes derived from dendritic cells activated CD4+ T-lymphocytes, which improved cardiac function following MI (217). On the other hand, restriction of dendritic cell activation and migration by inhibiting HMGB1 reduced infarct size as well (218).

Resident cardiac mast cells degranulate quickly after MI releasing histamine and pro-inflammatory mediators as TNF-α. Mast cells are increased in reperfused myocardium and are associated with upregulation of stem cell factor (SCF), which may expedite recruitment and homing of primitive bone marrow-derived cells contributing to the reparative phase (219). The most abundant protease in mast cell granules is tryptase, which induced synthesis of chemokines and cytokines to mediate leukocyte accumulation and angiogenesis (220) and was identified to regulate PKA activity modulating cardiomyocyte contractility after acute MI (221). Following MI-R injury, dual inhibition of mast cell and neutrophil-derived proteases reduced myocyte apoptosis and preserved cardiac function (222). Furthermore, inhibition of renin release from mast cells prevented activation of the renin–angiotensin system (RAS), reduced infarct size, and alleviated ventricular arrhythmias (223, 224).

### Reparative to Maturation Phase

#### Fibroblasts

Between days 3 and 7 following MI, activated cardiac fibroblasts proliferate, expand, and transdifferentiate into synthetic myofibroblasts preluding the reparative phase of cardiac repair due to the replacement of death cardiomyocytes by a matured fibrotic scar (225, 226). Interstitial fibroblasts surviving the ischemic event, or recruited bone marrow progenitor or epicardial-derived cells may undergo myofibroblast transdifferentiation (227) critically regulated by TGF-β. These myofibroblasts are marked by expression of α-smooth muscle actin (ASMA) and initiate the synthesis of ECM proteins and formation of neovessels (228). In the early post-ischemic period, fibroblasts may even modulate survival pathways in cardiomyocytes, affecting their susceptibility to myocardial ischemia (229). Moreover, myofibroblasts acquired anti-inflammatory properties following milk fat globule-epidermal growth factor (MFG-E)8-mediated engulfment of apoptotic cells (230).

Following MI, generation of active TGF-β in the injured myocardium triggers Smad activation in both fibroblasts and cardiomyocytes. Fibroblast-specific Smad3 seems essential for scar organization, since deficiency accentuated adverse remodeling with perturbed alignment of myofibroblast arrays in the infarct. In contrast, cardiomyocyte-specific Smad3 seems detrimental, as deficiency was associated with attenuated cardiomyocyte apoptosis without affecting infarct size (231). In addition, impaired TGF-β and Smad3 activation in fibroblasts depleted of the primary cilium resulted in decreased ECM protein levels and contractile function (232). Attenuating post-ischemic inflammation by administration of anti-inflammatory IL-10 improved cardiac remodeling and function by stimulation of fibroblast activation influenced by M2 macrophage polarization (233). Furthermore, increased cardiac hyaluronan synthesis contributed to post-ischemic infarct healing by promoting the myofibroblast response and supporting macrophage survival (234).

#### Neovascularization

Neovascularization is initiated by angiogenic growth factors followed by the recruitment of a muscular coat and formation of neoarterioles during scar maturation, which may stabilize infarct vasculature by inhibition of endothelial cell proliferation and vascular sprouting (235). Vascular endothelial growth factor (VEGF)-induced endothelial cell migration and proliferation cause hyperpermeable neovessel formation. Numerous factors, such as fibroblast growth factors (FGFs), TGF-β, MCP-1, IL-8, and IP-10 modulate angiogenesis, indicating its complexity. The post-ischemic increase in angiopoietin (Ang)-2 expression and reciprocal decrease in Ang-1 expression, suggested a predominant role for Ang-2 in the angiogenic response to MI (236). Furthermore, platelet-derived growth factor receptor (PDGFR) activation is involved in vasculature maturation, which promotes resolution of inflammation and stabilization of the myocardial scar (237). Following MI, cardiomyocyte-specific deficiency of HSPB1 resulted in decreased angiogenesis and collagen deposition with aggravated adverse remodeling and cardiac dysfunction as a result of enhanced leukocyte infiltration and expression of inflammatory cytokines (238).

### Resolution of the Post-ischemic Inflammatory Response

The acute transient inflammatory response finally promotes to a stage of tissue repair and scar formation (98, 225). Inhibition of chemokine and cytokine synthesis after they peaked is crucial for the repair process resulting in resolution of the inflammatory response (239). Although identifying the precise mechanisms controlling the switch from formation of pro-inflammatory to anti-inflammatory reparative pathways is an ongoing process, components of resolution include apoptosis, autophagy, necrosis, and formation of anti-inflammatory cytokines (240). Clearance of the neutrophilic infiltrate and removal of debris, inhibition of chemokine and cytokine synthesis, removal of the fibrin-based provisional matrix, and activation of fibroblasts and collagen deposition finally contribute to the myocardial reparative process and mature scar formation. This complex process of infiltration, differentiation, activation, and interaction of various cell types eventually provides accomplishment of complete infarct wound healing, which takes about 2 weeks in mice compared with 6 months in humans (241).

## Immunomodulatory Therapies in Clinical Practice

Although therapeutic interventions following myocardial infarction have been dramatically improved over the years, morbidity and mortality remain high. This could largely be explained by the practically lacking capability of the heart to regenerate (242), since differentiated cardiomyocytes predominantly lost their capacity to proliferate. In addition, myocardial wound healing occurs under continuous rhythmic contraction of the non-infarcted myocardium, thus under continuous mechanical stress (241). Treatment of patients to interfere in myocardial remodeling by healing of ischemic myocardium and preventing scar formation with concomitant preservation of cardiac function would be the ultimate goal. In the last years, cardiac regeneration has experienced a watershed moment, and it is believed that even in adult mammals, latent regeneration can be awakened. Combining stem cell-based methods with gene regulatory elements seems promising; however, correct electromechanical incorporation of transplanted cardiomyocytes remains a challenge as yet (243). In addition, combination of selected therapies might be promising exerting synergistic effects, for example, by inhibition of the early pro-inflammatory response with subsequent activation of the anti-inflammatory or the so-called reparative response. At the same time, effects regarding the non-inflammatory components, such as cardiomyocytes, platelet activation, microvascular obstruction, and coronary endothelial dysfunction should also be taken into account (244).

Already in 1973, the use of the anti-inflammatory agent corticosteroid for the first time showed limitation of myocardial infarct size in dogs (245). However, a subsequent clinical trial resulted in deleterious effects (246), possibly caused by delayed wound healing due to an impaired inflammatory response (247). This emphasizes the necessity of wound healing and illustrates the difficulty of counteracting maladaptive inflammatory pathways without interfering in benign wound healing endorsing inflammatory mediators to be notoriously pleiotropic. Current research focuses on targeted anti-inflammatory or immunomodulatory treatment, cardiac cell therapy and regeneration, mechanical therapeutic interventions, pharmacological therapies, and microRNA (miRNA)-targeted treatment. Below, anti-inflammatory and immunomodulatory therapies will be briefly highlighted. In addition, the selection of an appropriate animal model to ensure optimal translation of novel cardioprotective therapies from bench-to-bedside is of the utmost importance (248, 249), ideally allowing for an atherosclerotic phenotype and myocardial ischemia–reperfusion injury (250).

Although the immune response includes an essential part of the reparative myocardial wound healing process, no specific immunomodulatory treatments are available for patients on a large scale. Finished or discontinued clinical trials failed to translate promising results from experimental MI studies in animals toward clinical practice in human patients, although the post-ischemic inflammatory response remains amenable for therapeutic intervention where success depends on timing and topography emphasizing the necessity of real-time visualization of the extent and time course of cellular damage (251). In addition, right selection of subpopulations regarding distinct pathophysiologic perturbations and underlying mechanisms needs to be taken into account. Glucocorticoids were the first to be thought beneficial in post-ischemic remodeling. On the one hand, they attenuate the responses to danger signals during the acute inflammatory phase and reduce leukocyte infiltration; on the other hand, they are known to promote clearance of apoptotic cells and direct macrophages toward an anti-inflammatory phenotype. However, clinical studies showed conflicting results, and considering their broad actions on cell types involved in ischemic injury and effects on several molecular cascades, they are unattractive as a therapeutic option because of a wide variety of adverse events (252).

IL-1 signaling is suggested to play a crucial role in post-ischemic adverse remodeling and cardiac dysfunction, which is attenuated following IL-1 blockade with anakinra in animals (115), and represents a promising therapeutic approach following acute MI in patients (111, 112). Moreover, targeting the IL-1β innate immunity pathway with canakinumab reduced recurrent cardiovascular events in high-risk patients (253). Furthermore, administration of an IL-6R antagonist tocilizumab in patients suffering an acute coronary syndrome attenuated the inflammatory response (120). TNF-α blockade by etanercept reduced systemic inflammation but increased platelet activation following acute MI and was therefore concluded to be unfavorable (254). Targeted interventions regarding anti-inflammatory effects of IL-10 and TGF-β have not made it to a serious clinical trial yet. Trials with humanized recombinant antibody therapy directed against integrins (180) or the complement system (67, 255) failed to show positive effects. On the other hand, intracoronary nitrite following primary PCI for acute MI reduced neutrophil numbers and activation associated with a reduction in infarct size and microvascular obstruction (256). Immunomodulation with cyclosporine, which inhibits the opening of mitochondrial permeability-transition pores and exerts potent immunosuppressive effects, attenuated lethal myocardial injury (257), but did not preserve cardiac function following acute MI (258). Finally, anti-inflammatory therapy with colchicine following MI (259) as well as in patients with chronic coronary disease (260) led to reduced recurrent ischemic cardiovascular events.

## Conclusion

Acute myocardial infarction especially followed by reperfusion results in a complex post-ischemic inflammatory response encompassing a multifactorial and dynamic process consisting of overlapping inflammatory, reparative, and maturation phases. Therefore, MI-R injury is exceptionally amenable and, above all, suitable for immunomodulatory therapies. However, successful development and translation of future therapeutic strategies should focus on the sensitive balance between anti-inflammatory and pro-reparative effects and should be tested in translational research models exposed to clinically relevant pathophysiologic mechanisms and comorbidity.

## Author Contributions

NP wrote the manuscript. DA and PQ advised and edited the manuscript. All authors contributed to the article and approved the submitted version.

## Conflict of Interest

The authors declare that the research was conducted in the absence of any commercial or financial relationships that could be construed as a potential conflict of interest.
